# A Cut above the Rest: Characterization of the Assembly of a Large Viral Icosahedral Capsid

**DOI:** 10.3390/v12070725

**Published:** 2020-07-05

**Authors:** Erin R. Reilly, Milky K. Abajorga, Cory Kiser, Nurul Humaira Mohd Redzuan, Zein Haidar, Lily E. Adams, Randy Diaz, Juliana A. Pinzon, André O. Hudson, Lindsay W. Black, Ru-Ching Hsia, Susan T. Weintraub, Julie A. Thomas

**Affiliations:** 1 Thomas H. Gosnell School of Life Sciences, Rochester Institute of Technology, Rochester, NY 14623, USA; err4592@rit.edu (E.R.R.); mka8079@rit.edu (M.K.A.); clk9978@rit.edu (C.K.); nh5970@g.rit.edu (N.H.M.R.); zxh8923@g.rit.edu (Z.H.); lea4012@g.rit.edu (L.E.A.); rd3626@rit.edu (R.D.); jap4196@rit.edu (J.A.P.); Andre.Hudson@rit.edu (A.O.H.); 2 Department of Biochemistry and Molecular Biology, The University of Maryland School of Medicine, Baltimore, MD 21201, USA; lblack@som.umaryland.edu; 3 Department of Neural and Pain Sciences, University of Maryland Baltimore School of Dentistry, Baltimore, MD 21201, USA; RHsia@umaryland.edu; 4 Department of Biochemistry and Structural Biology, The University of Texas Health Science Center at San Antonio, TX 78229, USA; weintraub@uthscsa.edu

**Keywords:** *Salmonella*, giant phage, myovirus, head assembly, prohead protease, proteolytic maturation, mass spectrometry, transmission electron microscopy

## Abstract

The head of *Salmonella* virus SPN3US is composed of ~50 different proteins and is unusual because within its packaged genome there is a mass (>40 MDa) of ejection or E proteins that enter the *Salmonella* cell. The assembly mechanisms of this complex structure are poorly understood. Previous studies showed that eight proteins in the mature SPN3US head had been cleaved by the prohead protease. In this study, we present the characterization of SPN3US prohead protease mutants using transmission electron microscopy and mass spectrometry. In the absence of the prohead protease, SPN3US head formation was severely impeded and proheads accumulated on the *Salmonella* inner membrane. This impediment is indicative of proteolysis being necessary for the release and subsequent DNA packaging of proheads in the wild-type phage. Proteomic analyses of gp245- proheads that the normal proteolytic processing of head proteins had not occurred. Assays of a recombinant, truncated form of the protease found it was active, leading us to hypothesize that the *C*-terminal propeptide has a role in targeting the protease into the prohead core. Our findings provide new evidence regarding the essential role of proteolysis for correct head assembly in this remarkable parasite.

## 1. Introduction

The capsids, or heads, of all tailed phages have a thin protein shell of icosahedral symmetry that protects the dsDNA genome packaged to a density of ~500 mg/mL within it [1,2]. The outer shell is composed of the major capsid protein (MCP), and has at one vertex a turbine-shaped portal structure formed by 12 copies of the portal protein. The mature DNA-full head is the product of a remarkable series of steps that initiate with the assembly of a protein-only precursor structure, the procapsid or prohead which is typically spherical is shape [3]. Proheads undergo a series of maturation steps that create internal volume within the head and by doing so, allows the packaging of DNA into the head by the terminase protein [4,5]. Head maturation in tailed phages typically involves an expansion of the outer shell and loss of internal protein. In many phages, although not all, the loss of internal prohead material is mediated by a prohead protease that is incorporated into the prohead core during its assembly. 

It is well established that the MCP, portal, and terminase proteins of tailed phages all have conserved structural folds of such ancient origin they are also observed in the capsids of some eukaryotic viruses (e.g., herpesvirus) [6,7,8,9]. Tailed phage prohead proteases also have anciently derived folds, even sharing with the herpesvirus protease a catalytic mechanism that is dependent upon conserved serine and histidine catalytic residues [10,11,12]. The biochemical analyses of prohead proteases from a relatively small number of model phages (e.g., T4, Lambda, and HK97) support there being considerable diversity in substrate and cleavage specificities between different phages [13,14,15,16]. To date, all studied prohead proteases cleave a propeptide from the *N*-terminus of the MCP, and in doing so promote the structural transformation of the MCP to its mature form. In numbers of phages the prohead protease also cleaves internal prohead proteins, such as the scaffold protein [17]. Some phages have additional proteins that are substrates for their prohead protease. For instance, T4 has three prohead core proteins, the highly abundant scaffold protein gp22, as well as gp67 and gp68, [18,19] which undergo cleavage by the T4 prohead protease gp21 and then most of the resulting peptide fragments exit the head. T4 also has internal head proteins that are substrates for gp21 but remain in the mature head, the internal proteins IPI, IPII, and IPIII, and the ribosyltransferase, Alt [20,21,22,23]. These four proteins enter the host cell with the DNA at the onset of infection, earning them the name ejection, or E, proteins [20,21,22,23]. The IP proteins and Alt all have a short *N*-terminal propeptide (~10–20 amino acids) called a capsid targeting sequence (CTS) that interacts with the prohead core, thus mediating the incorporation of each protein into prohead [24]. During maturation, each CTS is removed via cleavage by gp21 and then exits the head, presumably via pores in the non-expanded capsid shell. 

Studies on model phages have shown there to be many variations in head assembly mechanisms between different phages. Based on these precedents, studies on phages with unusual head features will likely uncover novel mechanisms of head assembly. This is especially true for large phages with genomes >200 kb in length that are called “giant” or “jumbo” phages [25,26]. Giant phages require large capsids to package and protect their long genomes. Our research focuses on the giant *Salmonella enterica* myovirus SPN3US which has a 240 kb genome [27] packaged into its head. The large, T = 27 SPN3US head is comprised of ~50 different proteins that range in copy number by several orders of magnitude [28]. The most abundant head proteins are the major capsid, gp75 (~1560 copies) and the abundant E proteins, gp53 and gp54 (>600 copies) [29]. Our previous studies have demonstrated that numbers of the low abundance SPN3US head proteins are essential for viability. These include the five subunit virion RNA polymerase (vRNAP) and gp47 which are also expected to be E proteins [28,29]. However, the functions of the majority of SPN3US head proteins are unknown, as are the major steps by which the complex SPN3US head forms.

In this study, we present the characterization of two SPN3US prohead protease mutants using structural, proteomic and biochemical methods. The results demonstrated that the prohead protease is essential for SPN3US head formation and responsible for the cleavage of head proteins. In the absence of the protease, head maturation stalls and heads accumulate on the inside of the *Salmonella* inner membrane. Notably, our findings demonstrate that despite having overarching similarities to T4 head assembly and maturation, these processes in SPN3US have unique features that are different to any previously described phage.

## 2. Materials and Methods 

### 2.1. Isolation and Genome Sequencing of SPN3US Protease Mutant Phages 

SPN3US amber mutant phages were isolated using hydroxylamine mutagenesis as described previously [28]. Briefly, wild-type SPN3US was treated with 0.4 M hydroxylamine at 37 °C for ~24 h and this mixture was plated to obtain single plaques. Plaques underwent screening for detection of amber mutant candidates with a conditional-lethal phenotype (growth on permissive or suppressor hosts versus no growth on non-permissive or non-amber suppressing hosts). The bacterial strains used for these studies were provided by Dr. John Roth, University of California Davies (strains TT6675 (supD) and TT9079 (sup0); these strains and others in the Roth strain collection can be searched at the URL http://rothlab.ucdavis.edu/textStrainer), and Dr. Sherwood Casjens, University of Utah (strains UB-0018 (supF), UB-0015 (sup0)) and UB-0017 (supE)). Mutant candidates were propagated to high titer stocks that underwent reversion rate and complementation analyses. Mutant candidates that held true to an amber mutant phenotype, and were determined to be genetically different, underwent genome sequencing.

Genomic DNA was extracted from high titer stocks using the Norgen Phage DNA extraction kit following the manufacturer’s protocol. Mutant DNAs underwent a NexteraXT workflow and were sequenced on an Illumina MiSeq (2X200) at the University of Rochester Genomics Facility. Mutant genomes were assembled using DNASTAR SeqMan and single nucleotide polymorphisms (SNPs) were identified using SeqMan Pro. The wild-type (WT) phage genome (GenBank Accession JN641803.1) sequenced by Lee et al., [27] was used for reference-based alignments. 

### 2.2. Purification and TEM of SPN3US Amber Mutant Particles 

Liquid infections of SPN3US mutants in permissive or non-permissive bacteria were performed in LB + N broth at 33–34 °C at an MOI of 10 for 3 h. For infections in the non-permissive strain, at 25–30 min post infection cells were spun (5000 rpm, room temperature), and resuspended in fresh media to remove input phage. At the end of infection, cultures were treated with lysozyme (2 mg/mL) for 30 min at room temperature and underwent differential centrifugation to remove large debris and concentrate the remaining particles. The differential centrifugation involved a low speed spin (7000 g, 10 min, 4 °C) and a high speed spin (39,000 g, 40 min, 4 °C). Pellets were resuspended overnight in 4-(2-hydroxyethyl)-1-piperazineethanesulfonic acid (HEPES) buffer (pH 7.0) at 4 °C and then spun (8000 g, 10 min, 4 °C). The resulting supernatant immediately underwent purification by CsCl step (am59-supD), or sequential step and buoyant density gradient ultracentrifugation (am59-supF, am59-sup0, and am66-sup0), as described previously [28]. In several experiments, prior to purification the supernatants were treated with 1 mM (am59-sup0-a) or 2 mM (am59-sup0-b and am66-sup0) of the reversible cross-linker 3,3’-dithiobis(sulfosuccinimidyl propionate)(DTSSP) at 4 °C for 60 min. The DTSSP-treated supernatants were then quenched with Tris-HCl (50 mM, pH7.5) at 4 °C for 15 min and immediately loaded onto pre-poured CsCl step gradients. After CsCl gradient purification, gradient bands were harvested by side tube puncture and refractive indices measured on an Abbe refractometer. To remove CsCl, the harvested gradient bands were dialyzed against three changes of 0.2 Tm buffer (50 mM Tris-Cl, pH 7.5, 200 mM NaCl and 10 mM MgSO_4_) (3X 30 min, 4 °C). Purified particles were adsorbed to 400 mesh carbon-coated grids, negatively stained with 1% uranyl acetate and examined at 80.0 kV using a Tecnai T12 transmission electron microscope. See Supplementary Data for a description of preliminary experiments and further details. 

### 2.3. Mass Spectrometry Analyses of SPN3US Precursor Particles

Purified particles were boiled for 10 min in SDS sample buffer prior to electrophoresis on Criterion XT MOPS 12% or 4–12% gradient SDS-PAGE reducing gels (Bio- Rad) and proteins visualized by staining with Coomassie blue. Gel lanes were divided into six slices for GeLCMS analyses of each mutant; five individual bands were also excised from a gel lane of purified am59 grown under non-permissive conditions. Efforts were made to avoid transecting visible stained bands. No replicates of samples were analyzed. After de-staining, proteins in the gel slices were reduced with TCEP (tris(2-carboxyethyl)phosphine hydrochloride) and then alkylated with iodoacetamide in the dark before digestion with trypsin (Promega). HPLC-electrospray ionization-tandem mass spectrometry (HPLC-ESI-MS/MS) was performed on a Thermo Fisher LTQ Orbitrap Velos Pro mass spectrometer. Mascot (Matrix Science; London, UK) was used to search the MS files against a locally generated SPN3US and *Salmonella* protein database that had been concatenated with the SwissProt database (2012_11_170320; version 51.6). Subset searching of the Mascot output by X! Tandem, determination of probabilities of peptide assignments and protein identifications, and cross correlation of the Mascot and X! Tandem identifications were accomplished by Scaffold (Proteome Software). MS data files were either processed individually or the files for an entire gel lane were combined via the “MudPIT” option in Scaffold.

Detection of peptides generated by cleavage by the prohead protease was accomplished by database searching (Mascot and X! Tandem) using an enzyme specificity of “semi-trypsin” followed by inspection of peptide coverage in Scaffold (Proteome software). The results for identified proteins, numbers of unique peptides, total peptide spectrum matches, and sequence coverage for each experiment were exported from Scaffold with the following quality filters: Peptide, 95%; protein, 99.9%; minimum number of peptides, 2. An estimate of the relative abundance of SPN3US virion proteins was obtained by dividing the total number of peptide spectrum matches assigned to each protein (PSM) identified by MudPIT analyses by its molecular mass (PSM/M) as performed for SPN3US and related phages [29,30,31]. Our previous results have demonstrated that PSM/M provides a useful indicator of relative abundance of virion proteins. That is, proteins with similar PSM/M values are typically present in similar relative abundances in the virion; proteins with PSM/M ≤ 1 are likely to be present in only few copies, or even less than one copy, per virion. 

### 2.4. Cloning, Expression, and Purification of Truncated Forms of SPN3US gp245 

The cloning of the SPN3US protease gene SPN3US_0245 with an *N*-terminal six-histidine tag into the plasmid pHERD20T [32], was accomplished as described previously [29]. The SPN3US_0053 gene was cloned into the plasmid pHERD20T in the same manner [29]. SPN3US_0053 was amplified via PCR, cloned into pHERD20T using the *Nco*I and *Xba*I restriction sites. The codons for an *N*-terminal six-histidine tag were then added by site-directed mutagenesis. In addition, a truncated form of SPN3US_0245 (66T) ending at nucleotide 345 was synthesized as a Gblock (IDT) and cloned into pHERD20T. The 66T construct produces a gene product the same length of gp245 that would be expressed in mutant *245*am66 grown under non-permissive conditions. An additional 66T construct was synthesized in which the codon for the catalytic serine was mutated to an alanine codon (66T_S153A). Both 66T and 66T_S153A were synthesized with the codons for an *N*-terminal six-histidine tag and cloned into pHERD20T using the *Nco*I and *Bam*HI restriction sites. Proteins were expressed and purified, and protease assays performed as described previously [33].

### 2.5. Transmission Electron Microscopy (TEM) of SPN3US-Infected Salmonella

Cultures (exponential growth phase) of the TT9079 (sup0) strain were infected with WT SPN3US or amb59 at a multiplicity of infection (MOI) of ~10 in LB+N broth (33–34 °C, 180 rpm). Aliquots were taken at the indicated times, the cells pelleted (6 k, 3 min, RT) and resuspended immediately in PBS buffer with glutaraldehyde (1% final concentration). Cells were then repelleted and resuspended in PBS/glutaraldehyde solution and stored overnight at 4 °C. Cells were then pelleted and resuspended in 2% paraformaldehyde, 2.5% glutaraldehyde, 2 mM calcium chloride in 0.1 M piperazine-N,N′-bis(2-ethanesulfonic acid (PIPES) buffer (pH 7.4) for one hour. Fixed bacterial cells were washed and spun down in microfuge tubes and subsequently embedded in 2.5% low melting agarose. Agarose blocks containing bacteria were trimmed to 1 mm^3^ size and post-fixed with 1% osmium tetroxide, 1.5% potassium ferrocyanide in 0.1 M PIPES buffer for 60 min at 4 °C. Blocks were thoroughly rinsed in water and en bloc stained with 1% (*w*/*v*) uranyl acetate in water for 60 min. Specimens were washed, and dehydrated using serial graded ethanol solutions (30%, 50%, 70%, 90%, and 100% ethanol), and infiltrated and embedded in Spurs resin (Electron Microscopy Sciences, PA) following the manufacturer’s recommendations. Ultrathin sections ~70 nm thickness were cut on a Leica UC6 ultramicrotome (Leica Microsystems, Inc., Bannockburn, IL, USA) and collected onto copper grids and examined in a Tecnai T12 transmission electron microscope (Thermo Fisher Scientific, Formerly FEI. Co., Hillsboro, OR) operated at 80 kV. Digital images were acquired by using a bottom mount CCD camera (Advanced Microscopy Techniques, Corp, Woburn, MA, USA) and AMT600 software. 

### 2.6. Growth of SPN3US in Liquid Culture

Growth studies of SPN3US in *Salmonella* were performed in sterile Luria Bertani broth containing 2% nutrient broth (LB+N) that was supplemented with 2 mM CaCl_2_ and 2 mM MgCl_2_ at 34–35 °C. Single bacterial colonies were inoculated into 15 mL of LN+N broth and incubated with shaking (150 rpm) overnight (~16 hrs). Overnight cultures were subcultured (1:75 dilution) into fresh media and infected at an OD600 of 0.25–0.3 at an MOI of 0.1–1. Aliquots of each infection were taken at 5 and 10 min, and then every 10 min until 80 min post infection. Chloroform (2% final concentration) was added immediately after harvesting, if added. If chloroform was not added, samples were quickly centrifuged to pellet cells and the supernatant carefully removed. The supernatants were immediately diluted in SM buffer (50 mM Tris-HCl (pH 7.5), 8 mM magnesium sulfate, 100 mM sodium chloride, and 0.01% gelatin) and viable particles enumerated using spot tests initially, and then by plating the phage with the bacteria in the overlay. At least three biological replicates of each condition shown were performed. 

## 3. Results and Discussion 

### 3.1. Genome Sequencing of Mutant Phages Confirms the Essential Status of the SPN3US Protease 

Two SPN3US protease mutants [*245*(am59) and *245*(am66)] were identified during the creation of an SPN3US amber mutant collection. Both mutants propagated to high titer (typically ≥10^11^ pfu/mL) when plated on suppressor strains of *Salmonella* and had low reversion frequencies (typically ~10^-6^), when plated on non-permissive strains. Consistent with these observations, genome analyses showed each mutant to have a single nonsense, or amber, mutation in SPN3US_0245 (Table 1). These findings confirmed the mutants to be amber mutants, and their names adjusted to *245*(am59) and *245*(am66). These findings also established SPN3US_0245, whose gene product is the prohead protease gp245, as being essential. 

The analysis of the genomes of am59 and am66, also showed the mutant genomes had acquired additional mutations relative to the SPN3US genome deposited in GenBank (Accession JN641803.1), and two stocks of the WT phage employed in this study (Table 1, Table S1). The mutations in the *245*(am59) and *245*(am66) genomes that were within coding regions were categorized as either synonymous or non-synonymous mutations. Analyses of the protease mutants grown under amber suppressing conditions using TEM, SDS-PAGE and mass spectrometry (see below) indicated that the non-synonymous mutations did not have a significant impact on SPN3US head formation and maturation. Similarly, the single nucleotide insertion mutations in non-coding regions of the protease mutant genomes are also not expected to have an impact on the phenotypes of the mutants, as these mutations were derived from wild-type stocks of SPN3US (Table S1). We note that as these SPN3US mutants were isolated using a mutagenesis treatment that was previously used to create amber mutant collections of other phages, e.g., T4, that were mostly isolated prior to the routine use of whole genome sequencing. Based on our results, it seems likely that many of those other phage mutants also have background mutations in addition to their amber mutation.

### 3.2. SPN3US Protease Mutant 245(am59) Undergoes Normal Head Maturation under Amber Suppressing Conditions

Previous proteomic analyses of SPN3US showed that eight of its head proteins underwent post-translational modification. Of these, seven proteins were determined to have *N*-terminal propeptides, including the three most abundant proteins in the SPN3US head, the MCP gp75, and the two E proteins, gp53 and gp54 [29]. These analyses also determined that the predicted prohead protease, gp245 [33], had a *C*-terminal propeptide. Expression and purification of gp245 in vitro, showed that its *C*-terminal propeptide was removed via autocatalytic activity, supporting the assignment of gp245 as the prohead protease [29]. All eight SPN3US propeptides are cleaved *C*-terminal to a sequence motif reminiscent to that of T4 gp21 [29]. 

**Figure 1:**
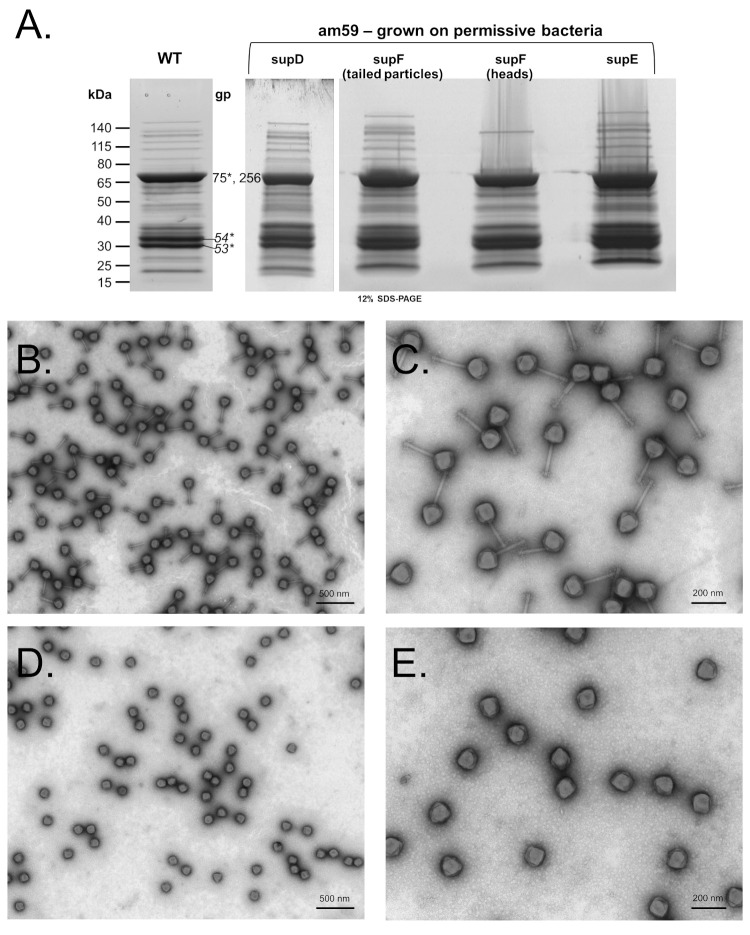
Characterization of *Salmonella* phage SPN3US amber mutant 245(am59) grown under amber suppressing conditions. (**A**) SDS-PAGE gels of purified mutant *245*(am59) grown under amber suppressing conditions compared to that of the wild-type phage (WT). The lanes containing *245*(am59) grown on supD and supF suppressor strains of *Salmonella* were used for mass spectrometry analyses. The mutant *245*(am59) grown on the supF strain produced large amounts of two different particles, as determined by TEM, those with wild-type phenotype (**B**,**C**) and tailless heads (**D**,**E**).

To determine if the normal SPN3US head cleavages occurred in a protease mutant grown under permissive conditions, mutant *245*(am59) was grown in glutamine, serine and tyrosine suppressor strains of *Salmonella* (supE, supD, and supF, respectively). The resulting particles were concentrated, purified and analyzed by SDS-PAGE, mass spectrometry and/or TEM (summarized in Table 2). In each CsCl gradient of purified *245*(am59) that had been grown under permissive conditions there was a band in a position that had a refractive index comparable to those from CsCl gradients of the WT phage (Table 2). The particles in each of the gradient bands had SDS-PAGE profiles (Figure 1A) and high titers (typically 10^11^–10^12^ pfu/mL) that were also comparable to those of the WT phage. These data led to the expectation that these *245*(am59) particles would be tailed. Supporting this expectation, TEM of the am59 from supF cells that banded in a gradient position with a refractive index close to that of the WT phage showed virions with WT phage morphology (Figure 1). The buoyant density gradient from the am59 supF infection also formed an additional band in a position in the CsCl gradient with a refractive index more similar to that obtained from DNA-full heads with no tails (Table 2) [28]. TEM of the particles from this am59 supF band showed DNA-full heads (Figure 1D and E). No second band was observed in the am59 supE gradient whose purification was performed at the same time as that of am59 grown in supF cells. We suspect that the replacement of glutamine with tyrosine at residue 115 in the protease may have impacted head maturation and resulted in the tailless heads in the am59-supF prep, although further research is required to test that hypothesis.

**Figure 2:**
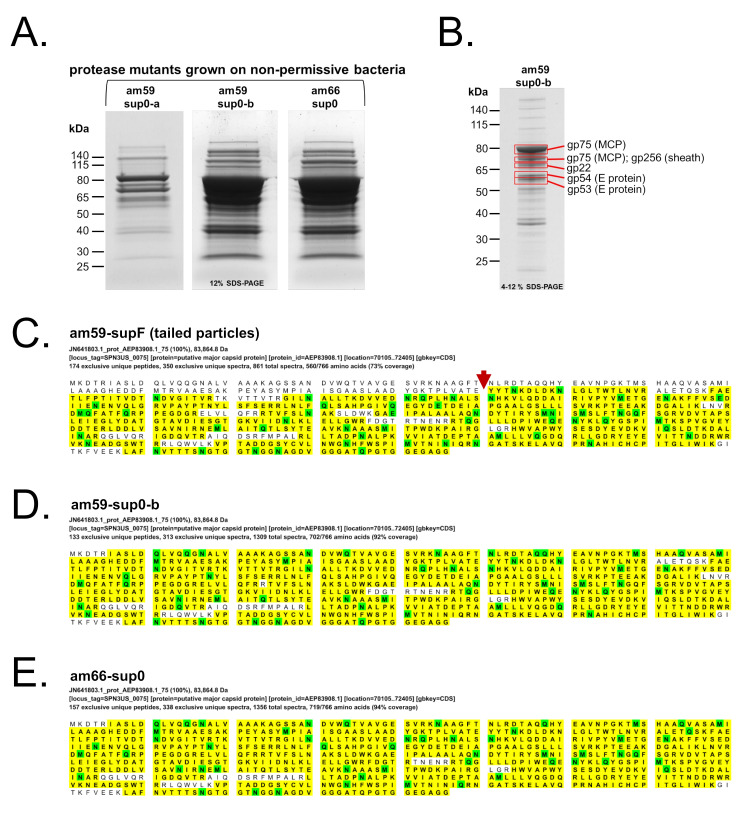
Characterization of *Salmonella* phage SPN3US protease mutants grown under non-amber suppressing conditions by SDS-PAGE and mass spectrometry. (**A**) SDS-PAGE gels of 245(am59) and 245(am66) grown on the non-amber suppressing (sup0) strain that were used for mass spectrometry analysis (Note, the am59-sup0-b and am66-sup0 lanes were heavily loaded in this gel so that low abundance proteins could be detected. A lower load of am59-sup0-b and am66-sup0 are shown electrophoresed through a 4–12% polyacrylamide gel in Figure S2). (**B**) SDS-PAGE showing the identity of the most abundant proteins in five gels slices of the major bands in purified samples of 245(am59) grown under non-permissive conditions. (**C**–**E**) Mass spectral protein sequence coverage of the SPN3US major capsid protein gp75 in protease mutants grown under amber suppressing and non-amber suppressing conditions. Amino-acids matched to a mass spectrum are shaded in yellow. Amino-acids marked in green potentially have a post-translational modification (e.g., phosphorylation). (**C**) The major capsid protein (MCP) showed evidence of normal cleavage by the prohead protease *C*-terminal to ATE-130 (red arrow) in the tailed particles formed by 245(am59) grown on a tyrosine amber suppressor (supF) strain (UB-0018). Normal cleavage of the MCP was not observed in protease mutants *245*(am59) (**D**) and *245*(am66) (**E**) grown under non-amber suppressing conditions (see also Table 3 and Table 4).

Analyses of the protein composition of the *245*(am59) particles derived from permissive infections indicated that normal head assembly and maturation had occurred. The SDS-PAGE profiles of all these *245*(am59) particles indicated that the major head proteins were cleaved comparably to those proteins in the WT phage [29] (e.g., Figure 1A). In addition, the mass spectrometry protein sequence coverage of *245*(am59) grown in supD or supF cells confirmed there had been cleavage of head proteins known to be cleaved in the WT phage (e.g., Figure 2C) [29]. 

The analyses of the tailed am59 particles grown in supF cells identified all the proteins found in the WT virion and three very low abundance proteins (gps 28, 156 and 199) (Table S2). Mass spectrometry of the DNA-full heads also from am59 grown in supF cells showed it to have comparable head protein composition to the tailed particles, with the exception that twelve proteins previously identified as tail or neck components were not identified (Table S2). Mass spectrometry of the am59 particles grown in supD cells identified 62 of 86 virion proteins (Table S2). The virion proteins not identified in am59-supD are all low abundance proteins in the WT phage (Table S2). Importantly, the virion proteins whose genes had gained non-synonymous mutations (gp47, gp61, gp145, gp168, gp239, and gp258), were all identified in the tailed *245*(am59) particles. In addition, the relative number of total identified peptide spectrum matches (PSMs) assigned for each of these proteins in the tailed am59 particles was comparable to their relative amounts in the WT phage (Table S2). Together, these data indicated that the other, non-amber, mutations in the am59 genome had no major impact on normal protease function when the mutant was grown under permissive conditions. These findings were important as they indicated that any differences in *245*(am59) grown under non-amber suppressing conditions would most likely be a consequence of the lack of suppression of the amber mutation in the gp245 gene. 

### 3.3. Immature SPN3US Proheads Accumulate in Protease Mutants Grown under Non-Amber Suppressing Conditions 

**Figure 3:**
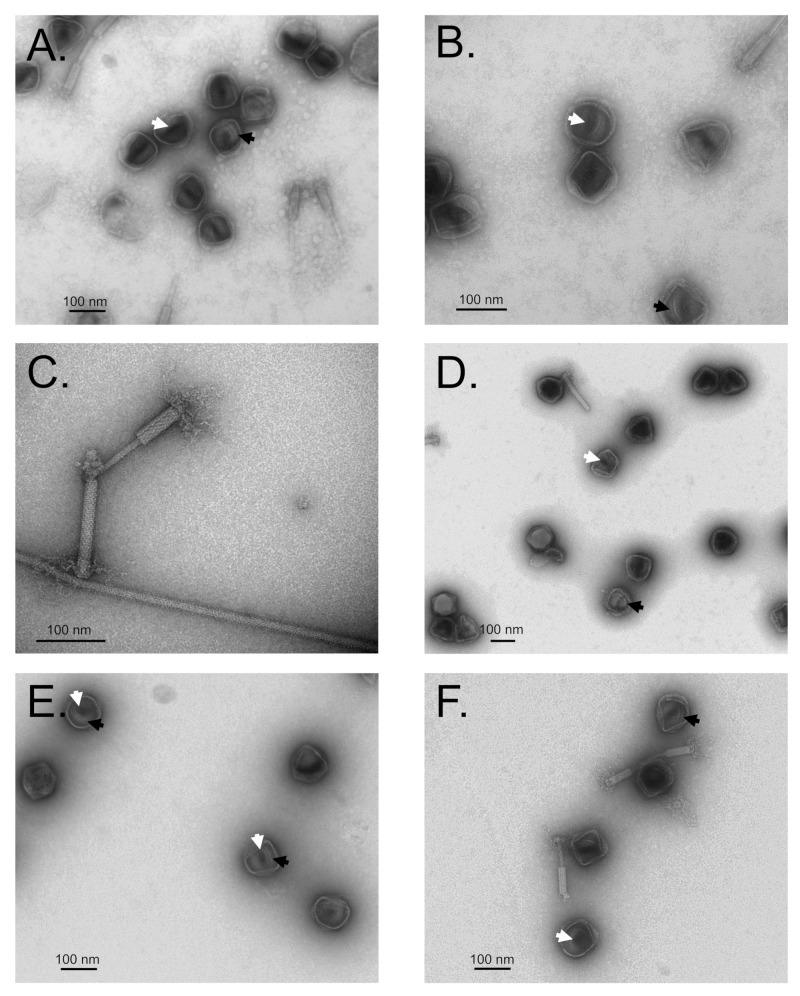
Transmission electron microscopy of *Salmonella* phage SPN3US protease mutants *245*(am59) (**A**–**D**) and *245*(am66) (**E**,**F**). Many precursor head particles and tails were observed. Numbers of precursor head particles were observed to have internal material (black arrow) that contained a central darkly staining region (white arrows). This darkly staining region was interpreted to not be DNA as there was no detectable DNA in these samples (see Figure S2). (**A**,**B**,**E**,**F**) Particles treated with DTSSP prior to purification (no extensive crosslinking of major head proteins was observed by SDS-PAGE). (**C**,**D**) Particles were treated with glutaraldehyde prior to purification. (**C**) A contractile tail apparently cross-linked to a flagellum via its baseplate/fibers.

To investigate the role of gp245 in SPN3US head formation, we studied infections of the SPN3US protease mutants grown under non-amber suppressing conditions after purification through two CsCl gradients (See Supplementary Data for preliminary experiments and further details). TEM of the particles from these infections revealed they were semi-purified and contained many proheads, some contracted tails and low quantities of flagella and other material (Figure 3, Figure S1). 

The proheads obtained from CsCl gradient purification of protease mutants grown under non-permissive conditions were heterogeneous, some particles were semi-angularized and some empty. However, a considerable number of proheads had internal material that was visible after the rupturing of the outer shell of these particles, likely on the grid during sample staining and/or drying (Figure 3). Many of these particles had a dark-colored central region (Figure 3), which we interpreted as representing a small hollow within the structure in which stain had pooled. Normally, intense dark staining within a mature, DNA-full, phage head is considered an artifact and referred to as positive staining [34]. We do not believe the intensely staining region within the SPN3US protease mutant proheads was due to positive staining because there was no detectable DNA in any of the CsCl gradient purified samples, as determined by agarose gel electrophoresis (Figure S2). 

Intriguingly, the TEM analyses of the protease mutant particles from non-permissive infections showed that the tails were not connected to the proheads. A large proportion of the tails were joined to one another, apparently by the exposed tail tube. In addition, the baseplate and fibers of several SPN3US tails were observed to be closely associated with flagella in samples that had been treated with glutaraldehyde (Figure 3C), consistent with the report by Lee et al. that SPN3US infection is flagella dependent [27].

### 3.4. SPN3US Proheads in Protease Mutant Infections Do Not Undergo Normal Proteolytic Maturation

Mass spectral analyses of purified protease mutants grown under non-amber suppressing conditions identified almost all the WT SPN3US virion proteins (Figure 2, Table S2). Several minor virion proteins (gp61, gp62, and gp245) were either not identified, or present in reduced peptide spectrum matches than in the WT phage (Table 3). Based on the proheads not being joined to the tails by TEM of the purified protease mutants, the absence of gp61 and gp62 support their being excellent candidates for head-tail joining proteins. Gp64, whose gene is near those of gp61 and gp62, was previously indicated as having a role in head-tail joining as a knockout in gp64 produced DNA-full heads [28]. 

Notably, the mass spectral protein coverage of the head proteins normally cleaved in the WT phage, such as gp75, gp53, and gp54 (all of which have long 124–130 residue propeptides), showed that these proteins had intact propeptides in particles from protease mutants grown under non-amber suppressing conditions (e.g., Figure 2D,E, Table 4). The absence, or extremely low, abundance of gp245 in these mutant particles supported the assignment of gp245 as the prohead protease (Table 3). The location of the protease in the WT SPN3US prohead is unknown. Based on the TEM observations, we speculate that the central region of the SPN3US prohead is a credible candidate for where gp245 is packaged early in head assembly, based on the protease mutant proheads apparently having no density in this region. If this speculation is correct, the location of the SPN3US protease within the prohead would be similar to that of T4, which was proposed to form a “kernel” within the prohead [35]. 

The mass spectral analyses of the particles derived from the two protease mutants grown under non-amber suppressing conditions identified a highly abundant protein, gp22 (Table 3, Table S2). The relative abundance of peptide spectrum matches identified for gp22 in those experiments was comparable to those for the major capsid protein, based on the mass spectrometry evidence (Table 3, Table S2). This was even in particles that had undergone dual gradient purification. In contrast gp22 was not identified in purified WT phage or DNA-full heads, with the exception of a low number of peptide spectrum matches identified in the *245*(am59) mutant grown in a serine suppressor and purified through a single CsCl gradient (am59-supD, Table S2). The identification of gp22 in samples containing large numbers of precursor head particles aligns with our proposed role for gp22 as a head scaffold or core protein that is removed during head maturation [36]. However, additional direct evidence is required to ascertain the function(s) of gp22.

### 3.5. The C-Terminus of the SPN3US Protease Likely Has a Role in Targeting the Protease into the Prohead 

The absence of normal head protein cleavages and the absence, or very low abundance, of gp245 in the purified protease mutant when grown under non-amber suppressing conditions, was intriguing. This is because the amber mutations in the protease gene of *245*(am59) and *245*(am66) would not completely knock out the expression of gp245 when grown under non-amber suppressing conditions. In addition, previous research had shown that an SPN3US mutant in gp47 had been able to package a *C*-terminal truncated form of a protein (gp47) into its head when grown under non-amber suppressing conditions. Gp47 is low abundance head protein that has a 111 residue *N*-terminal propeptide that is removed after cleavage by gp245) [29]. The particles produced in that infection were not viable despite having WT morphology and effectively the full complement of virion proteins. These results led us to hypothesize that the *N*-terminal propeptide of gp47 likely has a role in targeting the protein into the prohead, similar to the shorter capsid targeting propeptides of T4 head proteins [24,37].

**Figure 4:**
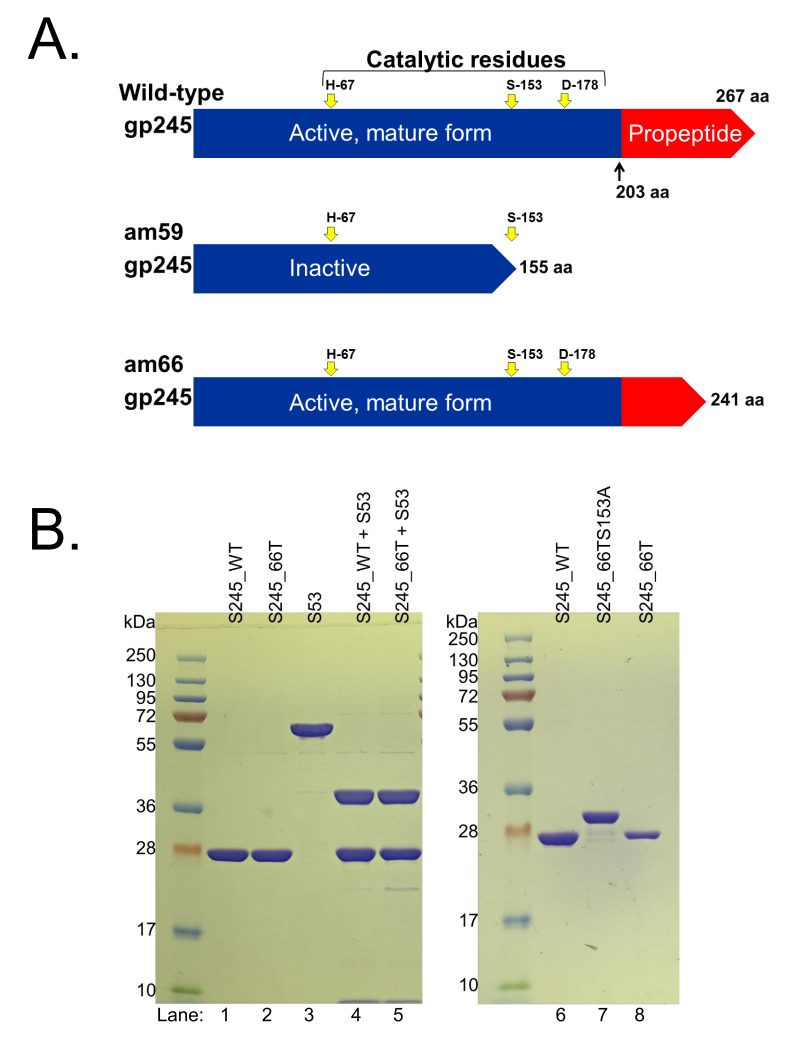
Characterization of enzymatic activity of the prohead protease in *Salmonella* phage SPN3US protease mutants. (**A**) Comparison of the wild-type (WT) SPN3US prohead protease, gp245, with the polypeptide chains expected to be formed during growth of amber mutants, *245*(am59) and *245*(am66), on amber suppressing bacteria. Note. The third catalytic residue would not be present in gp245 in *245*(am59) expressed under amber suppressing conditions, which would result in gp245 being inactive. (**B**) In vitro analysis of the SPN3US prohead protease. SDS-PAGE images: lanes 1 and 2, auto-cleavage products (removal of the propeptide) of recombinant WT gp245 and the truncated form (S245_66T) that is expected to be expressed in 245(am66) when grown under non- amber suppressing conditions; lane 3, SPN3US E protein gp53 (S53); lanes 4 and 5, cleavage of gp53 (S53) by both full-length gp245 (S245_WT) and the truncated form (S245_66T). SDS-PAGE image: lanes 6 and 8, auto-cleavage products of recombinant WT gp245 and the truncated variant (S245_66T); lane 7, a truncated mutant of gp245 (S245_66TS153A) that does not undergo auto-cleavage because the catalytic serine is not present.

Based on these precedents, the lack of incorporation of gp245 into the proheads of either protease mutant was especially curious since under non-amber suppressing conditions truncated forms of the protease would be still be produced (155 or 241 residues in *245*(am59) and *245*(am66), respectively) (Figure 4). The truncated form of gp245 from *245*(am59) is expected to be inactive as it would lack the third of three catalytic residues that are highly conserved in phage serine proteases [10,11,33] (Figure 4). By contrast, the truncated form of gp245 from *245*(am66) would be longer, such that it would retain 38 of the 64 residues that normally form the *C*-terminal propeptide [29]. Importantly, the truncated form of gp245 from *245*(am66) would retain all three of the catalytic residues essential for proteolysis in tailed phage prohead proteases (Figure 4) [10,11,33].

To assess the activity of gp245 produced by *245*(am66) when grown under non-amber suppressing conditions; we cloned the truncated form of the gene along with the codons for an *N*-terminal 6-His tag (S245_66T) into the expression vector, pHERD20T [32]. In addition, as an inactive enzyme control, we created a replicate length construct of S245_66T in which the catalytic serine was mutated to an alanine (S245_66TS153A). SDS-PAGE of the purified forms of these proteases showed that S245_66T migrated the same as WT gp245 whose maturation cleavage site had been confirmed previously by mass spectrometry [29]. By contrast, S245_66TS153A migrated slower under SDS-PAGE than either S245_66T or WT gp245 (Figure 4). These observations indicate that S245_66T had undergone autocatalytic cleavage in vitro. In addition, S245_66T was able to cleave the E protein gp53 (S53) comparably to the WT gp245 in vitro (Figure 4). 

These data support that the truncated form of the protease expressed in *245*(am66) grown under non-amber suppressing conditions was active. However, as noted above, the mass spectral data of the *245*(am66) prohead particles was more consistent with their having no protease incorporated into them. Our interpretation of these observations is that the *C*-terminal domain of the SPN3US protease likely contains a signal for packaging the protease into the prohead core during head assembly. 

### 3.6. SPN3US Proheads Assemble on the Inner Salmonella Membrane

**Figure 5:**
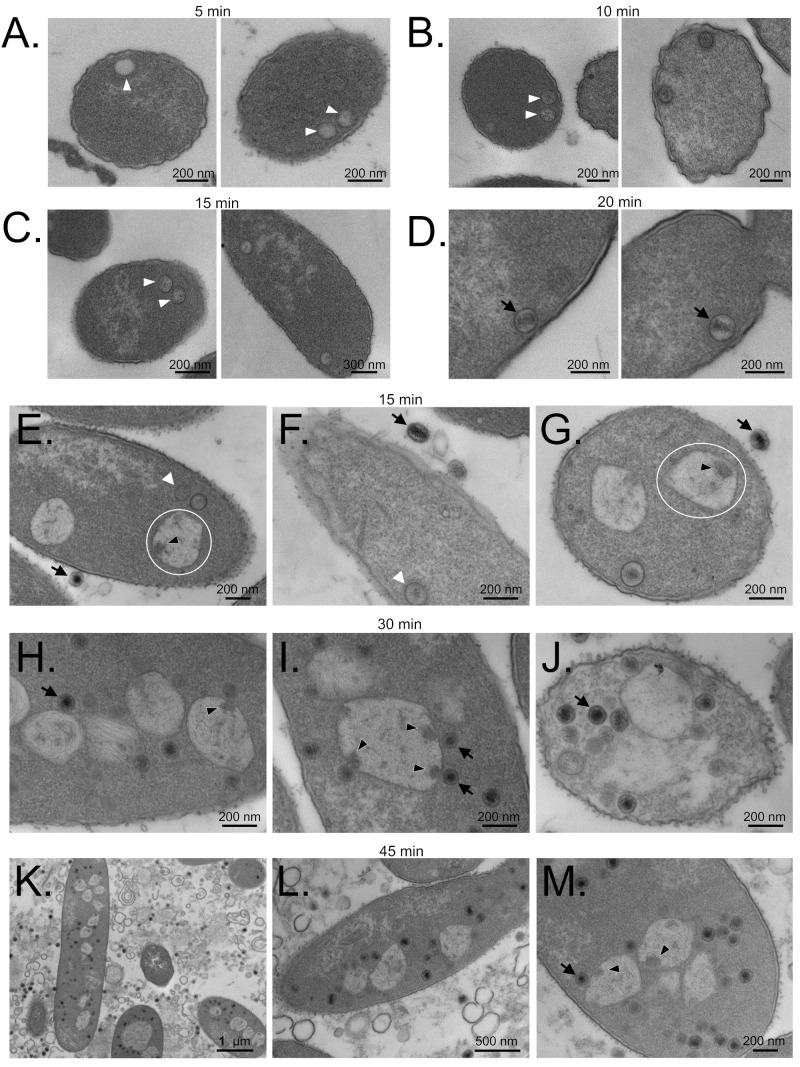
Transmission electron microscopy of thin sections of *Salmonella* Typhimurium cells infected with wild-type SPN3US. The times at which cells were harvested post infection are indicated. (**A**–**D**) Infection in which cells were harvested at 5, 10, 15, and 20 min after infection. (**E**–**M**) Infection in which cells were harvested at 15, 30, and 45 min after infection. Prohead particles are indicated with white arrowheads. Black arrows indicate examples of SPN3US heads (either within cells, D, or of input phage (**H**–**J**)) that have darkly staining material in their center. This material represents a condensed mass of ejection or E proteins within the packaged DNA. White circles indicate examples of lighter staining regions (**E**,**G**) called Giant phagE Masses (GEMs) which likely represents replicated phage DNA. Black arrowheads indicate darkly staining regions within several GEMs which may represent closely localized DNA replication, repair and recombination proteins.

To better understand the major steps in SPN3US head formation in vivo we performed TEM of thin section of the non-amber suppressing *Salmonella* strain TT9079 infected with the WT phage and *245*(am59). In WT phage infected cells, spherical prohead-like particles were observed in cells harvested as early as 5 and 10 min after infection (Figure 5A,B). At these time points, only 1–2 proheads were typically observed in the cells in which proheads could be observed (~30% of 202 cells). Early in infection all proheads that were observed appeared to be in close proximity to the *Salmonella* inner membrane (e.g., Figure 5A–C,E–G, Table S3). However, as infection proceeded many proheads were no longer observed to be close to the inner membrane (e.g., of 77 proheads examined from the 30 min time point, <50% were located near the inner membrane). In addition, as infection proceeded the numbers of proheads observed per cell increased. 

**Figure 6:**
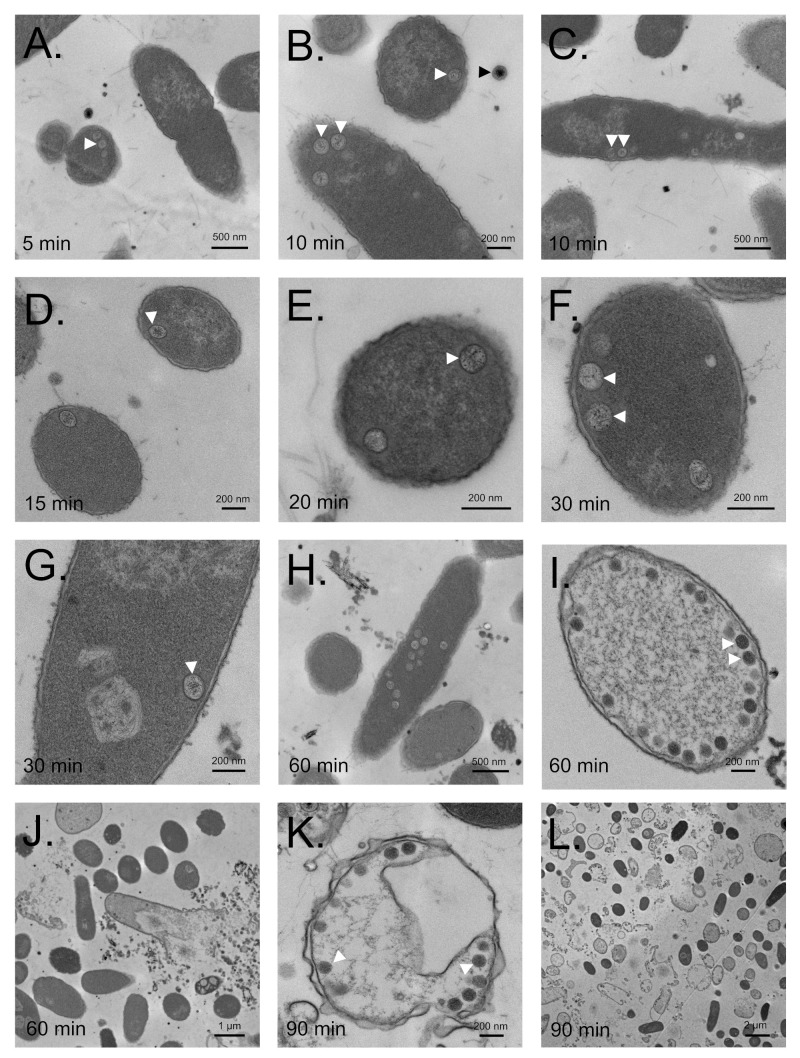
Transmission electron microscopy of thin sections of a non-amber suppressing strain of *Salmonella* infected with SPN3US protease mutant *245*(am59). The times at which cells were harvested after infection are indicated. (**A**–**G**,**I**,**K**) White arrowheads, precursor head particles close to the *Salmonella* inner membrane. (**B**) Black arrowhead, mature head of an input phage particle with darkly staining interior. (**H**,**I**,**K**) As infection proceeded increasing numbers of proheads were observed in many individual cells. Late in infection, many cells were observed to have lysed, as evidenced by cells with disrupted cell walls (**J**,**K**) and large amounts of cellular debris (**L**).

More conclusive evidence that SPN3US heads assemble on the *Salmonella* inner membrane was obtained via TEM of the non-amber suppressing strain TT9079 infected with *245*(am59) (Figure 6). In those cells, proheads remained in close proximity to the inner membrane throughout infection. That the mutant proheads were anchored to the inner membrane was inferred by the observation of many proheads still associated with the inner cell wall, even in cells whose cytoplasmic environment had been completely disrupted by lysis (e.g., Figure 6K). These data, in combination with the mass spectral analyses of SPN3US mutant proheads showing no evidence of proteolysis, support that the later steps in SPN3US head maturation, including prohead release from the host inner membrane, are in some way dependent upon proteolytic maturation by gp245. The determination that SPN3US proheads assemble on the inner *Salmonella* membrane represent an additional shared head assembly feature between SPN3US and T4 phage. T4 proheads have long been known to assemble on the *E. coli* inner membrane [38,39]. 

### 3.7. Maturation Results in Major Morphological Changes in WT SPN3US Proheads 

Comparison of thin sections from the WT and *245*(am59) SPN3US infections revealed additional variations in the proheads in each infection. Later in infection, many proheads in the WT SPN3US infection were observed to have dark-staining central regions with lighter exteriors (Figure 5D,H,J). This change was not observed in proheads in the am59 infection. The change in appearance of the WT proheads is likely caused by their having undergone proteolytic maturation and DNA packaging, or at least DNA packaging was well underway. That the particles represent mature, or close to mature, heads is inferred based on their similarity to the heads of input phages that had not infected a cell (e.g., Figure 5F,G). The darkly staining interior region in WT SPN3US heads likely represents a mass of its highly abundant E-proteins surrounded by lighter staining genomic DNA. Cryo-electron microscopy analyses support the presence of substantial amounts of protein within the SPN3US head. Elevated electron doses rendered images of SPN3US heads as “bubblegrams”, although these bubbles were not as consistently distributed as in the cylindrical “inner bodies” that have been observed in the heads of other giant phages—*Pseudomonas* phages ϕKZ, EL, Lin68, and 201ϕ2-1 (W. Wu, N. Cheng, A.C. Steven et al., manuscript in preparation) [40,41,42,43]. Overall, our data supports that the later steps in SPN3US head maturation, including prohead release from the host inner membrane and DNA packaging, are in some way linked to proteolysis. 

### 3.8. SPN3US Virion Formation and Infection Progress Rapidly

**Figure 7:**
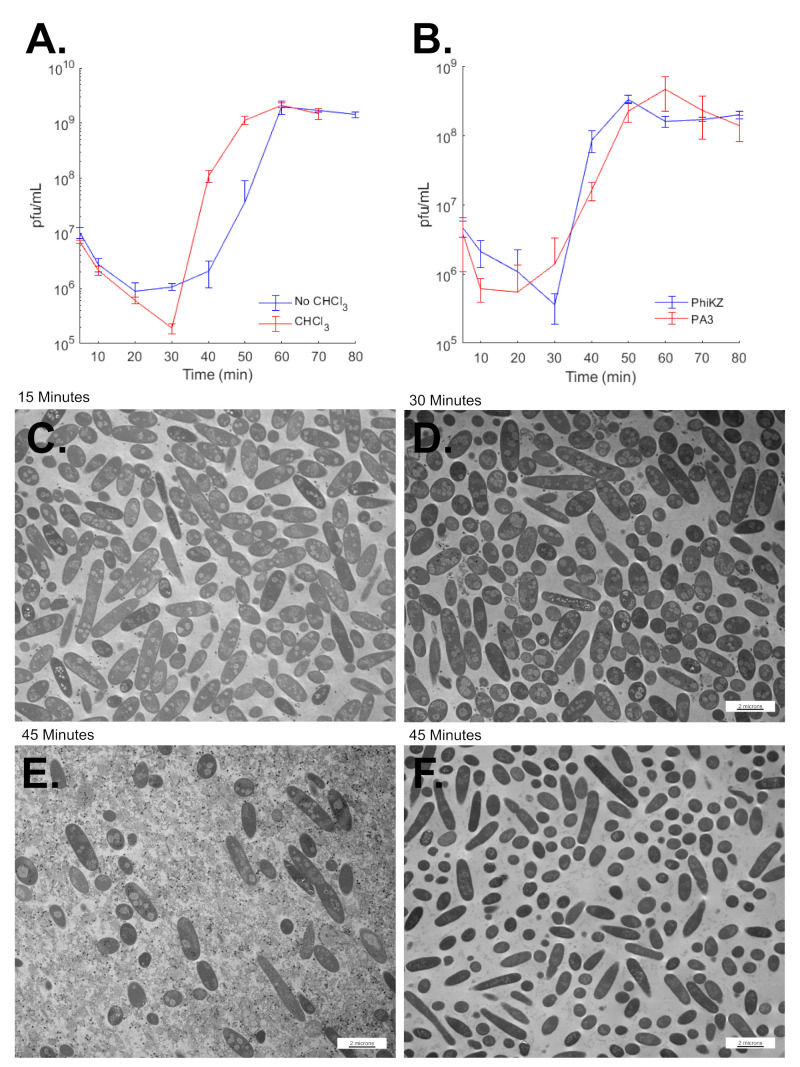
Growth curve and Transmission Electron Microscopy analyses of SPN3US infection of *Salmonella* in rich media. (**A**) Growth curve of SPN3US in liquid culture of *Salmonella* Typhimurium strain UB-0015. In one experiment, samples from replicate infections were treated with chloroform (red data series). In a separate experiment, samples from replicate infections were not treated with chloroform (blue data series). (**B**) Growth of *Pseudomonas aeruginosa* phages ϕKZ and ϕPA3 in liquid culture (samples from replicate infections were treated with chloroform immediately after sampling). The mean and standard deviation of three biological replicates are shown for each infection time point. Infections were at a multiplicity of infection of 0.1. (**C**–**F**) Transmission electron microscopy of thin sections of *Salmonella* Typhimurium cells at low magnification. Cells were infected with wild-type SPN3US (**C**–**E**). A control culture to which no phage was added was included in the experiment (**F**). Both infected and control cultures were from the same subculture. The times post infection at which cells were harvested for imaging are indicated. Cells at Time-0 are not shown.

The TEM of WT SPN3US-infected TT9079 cells sampled at 15, 30, and 45 min post infection indicated that the phage replication cycle was complete, or close to complete, by 45 min post infection. This is because, in that infection, many cells were observed to have lysed by 45 min post infection (Figure 7). To gain additional data on the overall time required for SPN3US head and virion assembly, we analyzed SPN3US infections with viability assays. These studies showed that SPN3US-infected cells begin to lyse between 40–50 min post infection when the *Salmonella* TT9079 strain was used (Figure 7). Cells lysed slightly earlier, at ~30 min post infection, in the UB-0015 strain that reaches exponential stage more rapidly than the TT9079 strain (Figure 7). In those infections, the average burst size for SPN3US was 117 in non-chloroform treated cells. 

These data indicate, that despite having a long genome and a virion of high complexity SPN3US is able to replicate and form viable virions in a comparable amount of time to tailed dsDNA phages with shorter genomes and less complex virions (based on total numbers of different proteins). This phenomenon is not unique to SPN3US, for example the giant *Bacillus* phage AR9 was found to have a similar replication time by Lavyesh et al. (2017) [44], as were giant phages ϕKZ and ϕPA3, when propagated under the same growth conditions as SPN3US (this study, Figure 7). 

### 3.9. SPN3US Infection Alters Salmonella Cell Morphology 

During our studies on the formation of SPN3US heads, we noted that SPN3US infection and replication had a considerable impact on *Salmonella* cells (Figure 5). In comparison to uninfected cells, infected cells appeared swollen and distorted, and their cytoplasm contained large, poorly staining regions (Figure 5 and Figure 7). These regions were non-uniform and roughly circular in appearance, and often several hundred of nanometers in diameter. The numbers of these poorly-stained regions varied between cells, but numerous SPN3US-infected cells were observed to have multiple regions, some >10 (e.g., Figure 7). We called these regions GEMs (Giant phagE Masses) as they were only observed in the SPN3US infected cells (Figure 7). GEMs potentially represent masses of DNA based on their similarity in appearance to images of known masses of DNA (“DNA-plasm”) in bacterial cells fixed with osmium tetroxide and stained with uranyl acetate [45,46]. Many GEMs were observed to have a fine fiber-like network, similar to those observed in bacterial nucleoids (e.g., Figures 10 and 12 in Robinow and Kellenberger [45]). We hypothesize that GEMs represent large masses of concatemeric SPN3US DNA, especially as many SPN3US proheads were observed near the exterior of GEMs late in infection of the WT phage when DNA packaging is likely to be occurring. However, further studies are required to test this hypothesis and to understand the composition and function of GEMs. 

## 4. Conclusions

**Figure 8:**
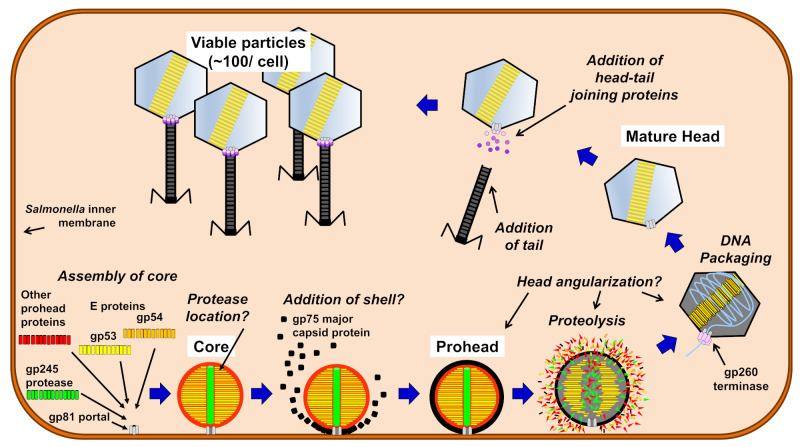
Model of *Salmonella* phage SPN3US head assembly and maturation.

The mechanisms by which giant phage heads form their large and complex heads are poorly understood. Previous studies have shown that giant *Salmonella* phage SPN3US has highly diverged counterparts to several major T4 head morphogenesis proteins (e.g., protease, terminase, portal, and major capsid protein) which suggest there are likely some shared head assembly mechanisms between the two phages [29]. In addition, previous mass spectral analyses of the SPN3US virion had shown that proteolysis must cause a major remodeling of the capsid since long propeptides were removed from several high abundance head proteins, including the major capsid protein [29,30,31]. In the current study, we characterized SPN3US prohead protease mutants using TEM and mass spectrometry. The evidence obtained from their analyses confirm that the SPN3US prohead protease, gp245 is responsible for the removal of the propeptides from cleaved head proteins. Our studies also confirm that gp245 has a pivotal role in SPN3US head formation, a model of which is shown in Figure 8.

Our findings provide additional support to our previous studies that indicated SPN3US head assembly has shared ancestry with that of T4. For instance, proheads in protease mutant infections remained anchored to the *Salmonella* inner membrane, and were unable to undergo DNA packaging. However, our data also highlight that SPN3US head assembly and maturation has numbers of unique steps relative to those of other characterized phages, including T4. For instance, our analyses revealed that in the absence of proteolysis, normal head assembly is aborted. However, in vitro assays of a truncated form of the protease that would be expressed in one of the protease mutants (missing 26 of the 64 residues of the *C*-terminal propeptide) were active. Based on these results, we propose this propeptide has a role in targeting the SPN3US protease into the center of the prohead early in assembly. In addition, we identified a highly abundant protein, gp22, in purified samples of the protease mutants confirmed to have a high concentration of proheads by TEM. This observation is consistent with gp22 having a role in head assembly, although further studies are required to elucidate fully its function(s). Our data also support that gp61 and gp62, with gp64, all have roles in head-tailing joining. Lastly, we observed in SPN3US-infected *Salmonella* cells multiple poorly staining regions which we believe represent masses of concatemeric DNA that are produced by phage-encoded proteins and are the substrate for DNA packaging. These observations indicate that further studies are needed to unravel all the steps required to form the mature SPN3US head and that such research is almost certain to identify further novel features of giant phage head assembly and maturation. 

## Figures and Tables

**Table 1:** Mutations identified by genome sequencing of the SPN3US amber mutants am59 and am66. Single nucleotide insertions in non-coding regions also identified in stocks of wild-type SPN3US (see Table S1) are indicated with an asterisk.

Mutant	Reference Position ^1^	Gene Name	DNA Change ^2,3,4^	Amino Acid Change ^5^	Impact	Function/Comment
**am59**	29457	SPN3US_0034	c.444G>A	p.Q148Q	Synonymous	Non-virion RNAP β
	46070	SPN3US_0047	c.1172G>A	p.G391D	Non Synonymous	Low abundance head protein
	59923	SPN3US_0061	c.211G>A	p.V71M	Non Synonymous	Putative neck protein
	85238	SPN3US_0086	c.261A>G	p.E87E	Synonymous	Non-virion protein
	114280	SPN3US_0135	c.222C>T	p.D74D	Synonymous	Non-virion protein
	122374	SPN3US_0145	c.857C>T	p.P286L	Non Synonymous	Low abundance head protein
	147028	SPN3US_0168	c.3692T>C	p.L1231P	Non Synonymous	Tail fiber protein
	186363	SPN3US_0213	c.187C>T	p.R63C	Non Synonymous	Non-virion protein
	210826	SPN3US_0239	c.1502C>T	p.A501V	Non Synonymous	Tape measure protein
	**221150**	**SPN3US_0245**	**c.343C>T**	**p.Q115.**	**Nonsense**	**Prohead protease**
	230553	SPN3US_0258	c.395C>T	p.T132M	Non Synonymous	Tail or neck protein
	239933 *		g.239933insT			Mutation in non-coding region
**am66**	77962 *		g.77962insT			Mutation in non-coding region
	87715 *		g.87715insC			Mutation in non-coding region
	93451 *		g.93451insT			Mutation in non-coding region
	135828	SPN3US_0156	c.404G>A	p.G135E	Non-synonymous	Non-virion protein
	180411	SPN3US_0204	c.395C>T	p.A132V	Non-synonymous	Non-virion protein
	200944 *		g.200944insT			Mutation in non-coding region
	**221528**	**SPN3US_0245**	**c.721C>T**	**p.Q241.**	**Nonsense**	**Prohead protease**
	223871	SPN3US_0250	c.70C>A	p.R24S	Non-synonymous	Non-virion protein
	239933 *		g.239933insT			Mutation in non-coding region

^1^ Reference position, refers to the nucleotide sequence position in GenBank Accession JN641803. ^2^ A “c.” prefix, followed by coordinates taken from the ORF, denotes a change in a coding sequence feature. ^3^ A “g.” prefix followed by genomic coordinates denotes a change in an intergenic region. ^4^ “ins” refers to single nucleotide insertion. ^5^ A “p.” prefix, followed by coordinates taken from the protein, denotes a change in a protein feature.

**Table 2:** Summary of SPN3US and *Salmonella* proteins identified by mass spectrometry in protease mutants grown under amber suppressing and non-amber suppressing conditions. ND, not determined. See Table S2 for total peptide spectrum matches for each protein identified in each experiment.

					SPN3US		*Salmonella*		
SPN3US Phage/Mutant	Experiment Abbreviation	Purification ^1^	Refractive Index	Buoyant Density, g/mL	No. of Proteins Identified	Total PSM ^2^	No. of Proteins Identified	Total PSM ^2^	*Salmonella*/SPN3US PSM ^2^, %
**Wild-type ^3^**	WT	CsCl step gradient ^4^	1.3735 (Lower, “junk” band = 1.3695/1.3694)	1.42 (1.38)	86	8513	28	470	5.5
***245*(am59)**	am59-supD	CsCl step gradient ^4^	1.3725	1.41	62	2638	42	410	15.54
	am59-supF (tailed)	CsCl step and buoyant density gradients ^5^	1.3735	1.42	88	9689	20	72	0.74
	am59-supF (heads)	CsCl step and buoyant density gradients ^5^	1.3749	1.43	74	9214	16	80	0.87
	am59-sup0-D	Differential concentration only	NA	NA	90	4018	188	2232	55.55
	am59-sup0-SB	CsCl step bottom band ^4^	1.3685	1.37	66		186		
	am59-sup0-ST	CsCl step top band ^4^	1.3636	1.32	ND	ND	ND	ND	ND
	am59-sup0-a	CsCl step and buoyant density gradients ^5^	1.3650	1.33	75	4405	36	361	8.20
	am59-sup0-b	CsCl step and buoyant density gradients ^5^	1.3640	1.32	101	11326	80	857	7.57
	am59-sup0-glut	CsCl step and buoyant density gradients ^6^	1.3640	1.32	ND	ND	ND	ND	ND
***245*(am66)**	am66-sup0	CsCl step and buoyant density gradients ^5^	1.3638	1.32	113	11102	83	640	5.76

^1^ Purification by CsCl gradient centrifugation occurred after concentration by differential centrifugation. ^2^ PSM, peptide spectrum matches. ^3^ The wild-type SPN3US proteome was published previously [29]. ^4^ Purification by CsCl step gradient centrifugation. ^5^ Purification by CsCl step and buoyant density gradient centrifugation. ^6^ Purification by CsCl step and buoyant density gradient centrifugation after treatment with glutaraldehyde.

**Table 3:** Highly abundant proteins identified by mass spectrometry in SPN3US protease mutants grown under amber suppressing or non-amber suppressing conditions. Several low abundance proteins of interest due to their reduction under non-amber suppressing conditions are also included. Total peptide spectrum matches adjusted by molecular mass (PSM/M) are provided. Mass values of processed proteins were used for particles from amber suppressing infections. Intact masses were used for proteins from particles from non-amber suppressing infections, because no cleavage by the prohead protease was observed for those proteins. See Table S2 for all proteins and the total assigned peptide spectrum matches for each protein in each experiment.

			Amber Suppressing Growth Condition (PSM/M) ^1^				Non-Amber suppressing Growth Conditions (PSM/M) ^1^			
gp	Function	Mass, kDa (Proc. ^2^)	WT	am59-supD	am59-supF (Tailed)	am59-supF (Heads)	am59-sup0-D	am59-sup0-a	am59-sup0-b	am66-sup0
***Highly abundant proteins***										
75	Major capsid	83.9 (70.4)	22.61	9.26	22.78	33.51	10.60	15.11	33.06	31.33
22	Scaffold candidate	67.1	0.00	0.40	0.00	0.00	8.91	16.99	21.33	26.87
256	Tail sheath	75.7	8.84	3.36	8.27	0.94	2.60	3.71	9.82	11.72
53	E protein	45.2 (31.5)	21.02	5.65	24.86	29.68	9.98	5.55	14.12	10.53
54	E protein	45.1 (31.9)	20.16	3.64	21.00	29.31	7.27	5.01	10.22	9.02
***Low abundance proteins***										
81	Portal	100.2 (72.3)	1.29	0.39	1.22	2.09	0.06	0.11	0.76	0.82
245	Prohead protease	30.7 (23.4)	1.07	0.13	1.11	1.11	0.00	0.00	0.07	0.07
61	Possible neck	58.3	0.60	0.10	0.50	0.00	0.00	0.00	0.00	0.00
62	Possible neck	52.0	0.85	0.19	0.77	0.04	0.00	0.00	0.00	0.00
64	Possible neck	48.9	0.41	0.00	0.41	0.00	0.00	0.04	0.37	0.20

^1^ PSM/M, Total assigned peptide spectrum matches/mass. ^2^ Predicted mass of mature form, after processing by the prohead protease.

**Table 4:** Peptides from *Salmonella* phage SPN3US head proteins identified by mass spectrometry of prohead protease mutants grown under non-amber suppressing conditions showing an absence of cleavage at their normal prohead protease cleavage sites.

Protein	Cleavage Site	Residues	Peptide Sequence ^1^	No. of Peptide Spectrum Matches	
				am59-sup0-b	am66-sup0
gp75, MCP	ATE-130	124–150	TPLV**ATE**YYTNKDLDKNLGLTWTLNVR	53	60
		124–139	TPLV**ATE**YYTNKDLDK	14	14
		124–135	TPLVATEYYTNK	12	10
		94–135	VAAESAKPEYASYMPIAISGAASLAADYGKTPLV**ATE**YYTNK	4	9
		94–150	VAAESAKPEYASYMPIAISGAASLAADYGKTPLV**ATE**YYTNKDLDKNLGLTWTLNVR	0	5
		94–139	VAAESAKPEYASYMPIAISGAASLAADYGKTPLV**ATE**YYTNKDLDK	0	1
		124–148	TPLV**ATE**YYTNKDLDKNLGLTWTLN	1	1
		124–145	TPLV**ATE**YYTNKDLDKNLGLTW	1	1
		124–138	TPLV**ATE**YYTNKDLD	1	1
		124–136	TPLV**ATE**YYTNKD	1	1
gp81, Portal	ATE-161 (expected maturation Site: AQE-254)	136–164	GASPVLLISDTGFDELFGLKPSV**ATE**SLR	1	2
		136–165	GASPVLLISDTGFDELFGLKPSV**ATE**SLRR	1	2
gp53, E protein	AQE-125	122–137	A**AQE**GWKETLKDLFER	10	5
		122–132	A**AQE**GWKETLK	2	1

^1^, Bold, residues which conform to the SPN3US protease cleavage motif (AXE). The protease cleaves *C*-terminal to these residues in the WT phage.

## Supplementary Materials

The following are available online at https://www.mdpi.com/1999-4915/12/7/725/s1, Figure S1: Transmission electron microscopy of *Salmonella* phage SPN3US protease mutants grown under non amber suppressing conditions during purification. (A-F) 2*45*(am59) and (G-I) 2*45*(am66). (A and B) Differentially concentrated 2*45*(am59), (C) Lower band from a CsCl step gradient of 2*45*(am59), (D and E) CsCl buoyant density top band of 2*45*(am59), (F) CsCl buoyant density bottom band of 2*45*(am59), (G) CsCl buoyant density top band of 2*45*(am66), and (H and I) CsCl buoyant density bottom band of 2*45*(am66)., Figure S2: SDS-PAGE and DNA gels of purified *Salmonella* phage SPN3US protease mutants *245*(am59) and *245*(am66) grown under non-amber suppressing conditions. (A) Example CsCl step gradient banding profile of *245*(am59). (B) SDS-PAGE of the mutant *245*(am59) in comparison to the profile produced by the mutant purified after growth under amber suppressing conditions (am59-supF). Gel polyacrylamide concentration was 12%. (C) Example CsCl buoyant density gradient banding profile of *245*(am59). (D) SDS-PAGE of buoyant density gradient bands from *245*(am59) and *245*(am66), compared to purified wild-type (WT) phage. Gel polyacrylamide concentration was 4–12%. (E and F) Agarose gel electrophoresis of CsCl purified *245*(am59) and *245*(am66) from (B) and (D) versus WT phage and am107 (am107 forms DNA full heads by TEM, data not shown). Gels contained 1% agarose in TAE buffer. Key: D, sample underwent differential concentration; DD, differentially concentrated sample underwent treatment with DTSSP; B, sample underwent CsCl buoyant density purification; BT, buoyant density gradient top band, BB, buoyant density gradient bottom band; M, Gene Ruler 1 kb DNA ladder (Thermo Scientific), 1 µg of total DNA was loaded per lane. Table S1: Single Nucleotide Polymorphism (SNP) report from genome sequencing of two SPN3US wild-type phage stocks and two SPN3US prohead protease amber mutants. The report was generated by a SeqMan NGen assembly (DNASTAR), Table S2: Identification of proteins by mass spectrometry in SPN3US protease mutants grown under amber suppressing and non-amber suppressing conditions, Table S3: Numbers of proheads observed in TEM micrographs of thin sections of *Salmonella* cells infected with wild-type SPN3US.
