# Supplementary material for: A Cut above the Rest: Characterization of the Assembly of a Large Viral Icosahedral Capsid
Source: Viruses. 2020 Jul 5;12(7):725. doi: 10.3390/v12070725 (PMC7411985; doi:10.3390/v12070725)
Supplement: Supplementary file 1 [file viruses-12-00725-s001.zip › Supplementary/Reilly_et_al_Supplementary_Data_Figures.pdf]

Supplementary Data and Figures.

A Cut Above The Rest: Characterization of the Assembly of a Large Viral Icosahedral Capsid

Erin R. Reilly[1#], Milky K. Abajorga[1#], Cory Kiser[1], Nurul Humaira Mohd Redzuan[1], Zein Haidar[1], Lily E. Adams[1], Randy Diaz[1], Juliana A. Pinzon[1], André O. Hudson[1], Lindsay W. Black[2], Ru-Ching Hsia[3], Susan T. Weintraub[4], and Julie A. Thomas[1*]

[1] Thomas H. Gosnell School of Life Sciences, Rochester Institute of Technology, Rochester, New York, United States of America.

[2] Department of Biochemistry and Molecular Biology, The University of Maryland School of Dentistry, Baltimore, Maryland, United States of America.

[3] Department of Neural and Pain Sciences, University of Maryland Baltimore, School of Dentistry, Baltimore, Maryland, United States of America.

[4] Department of Biochemistry and Structural Biology, The University of Texas Health Science Center at San Antonio, Texas, United States of America.

Supplementary Data

Preliminary analyses of SPN3US mutant *245*(am59) grown under non-amber suppressing conditions

To investigate the potential of the protease mutants grown under non-amber suppressing conditions for studies on SPN3US head formation, we conducted preliminary experiments on the *245*(am59) grown under non-amber suppressing conditions. TEM of particles that had been concentrated by differential centrifugation (am59-sup0-D) revealed spherical-like objects that were potential proheads amongst a large amount of cell debris, many flagella, and tails (data not shown). Five-fold dilution of this sample and treatment with 1 % glutaraldehyde revealed the presence of thick-walled proheads (Fig.S1). Many of the proheads had an outer shell that was disrupted, suggesting they were fragile. Mass spectrometry analyses of the am59-sup0-D sample identified a large number of *Salmonella* proteins, as would be expected for an unpurified sample (Table 2, Table S2). Flagellin was the most abundant *Salmonella* protein (Table 2, Table S2). Despite the contaminating host proteins, the most abundant proteins in the sample were SPN3US proteins, with almost the full complement of SPN3US virion proteins identified (Table S2).

Notably, gp245, the predicted prohead protease, was not identified in this experiment and the mass spectral protein sequence coverage of SPN3US head proteins that are normally cleaved in the WT phage showed no evidence of their cleavage in this sample (data not shown). The lack of proteolytic processing of SPN3US head proteins was consistent with the altered SDS-PAGE profile of this sample relative to the WT phage and the presence of immature head particles in it observed by TEM. The results of these preliminary experiments on am59 supported the assignment of gp245 as the SPN3US prohead protease, and encouraged us to undertake further analyses of protease mutant samples.

Purification of SPN3US protease mutants grown under non-amber suppressing conditions

To further investigate the role of gp245 in SPN3US head formation, we studied three infections of the SPN3US protease mutants under non-amber suppressing conditions [two infections of *245*(am59) (am59-sup0-a, am59-sup0-b) and one of *245*(am59) (am66-sup0)]. These samples were differentially concentrated, treated with 1 mM (am59-sup0-a) or 2 mM (am59-sup0-b and am66-sup0) of DTSSP, and purified through two CsCl gradients (e.g., Fig. S2). TEM of the three samples revealed they were semi-purified and contained many prohead-like particles (Fig. S1). The first CsCl step gradient purification of the purified protease mutant samples produced two main bands. SDS-PAGE of these bands showed the upper band to have a profile was consistent with the presence of unprocessed head proteins in that sample (Fig. 3B), as expected from the preliminary experiment. TEM of the lower step gradient band showed large amounts of flagella and cell debris (not shown). These data indicated that the upper band was of interest for further analyses.

Subsequent buoyant density gradient purification of the upper band from the CsCl step gradient produced semi-purified SPN3US proheads, as determined by TEM of the am59-sup0-a sample (Fig. S1A, B). The am59-sup0-b and am66-sup0 infections were performed simultaneously, each with a starting culture volume double that used for the am59-sup0-a infection. The more concentrated am59-sup0-b and am66-sup0 samples facilitated the observation of two bands extremely close to one another in the buoyant density gradient (Fig. S2C). The two bands in each gradient were harvested separately, as much as was feasible, and found to have very similar refractive indices (Table 2). SDS-PAGE of dialyzed samples from the two bands showed that the top bands (BT) contained less gp53 and gp54, and more flagella, than the lower band (Fig. S2D). TEM supported there being higher numbers of flagella in the top band, as well as proheads (many appearing empty with completely broken shells), tails, and material that appeared to be from disrupted head particles (not shown). TEM of the bottom bands (BB) from the buoyant density gradient showed it also contained many proheads, some contracted tails (many joined by the exposed tail tube) and lower quantities of flagella and other material than in the top band (data not shown).

Figure S1. Transmission electron microscopy of *Salmonella* phage SPN3US protease mutants grown under non amber suppressing conditions during purification. (A-F) Samples of 2*45*(am59) and (G-I) 2*45*(am66). (A and B) Differentially concentrated 2*45*(am59), (C) Lower band from a CsCl step gradient of 2*45*(am59), (D and E) CsCl buoyant density top band of 2*45*(am59), (F) CsCl buoyant density bottom band of 2*45*(am59), (G) CsCl buoyant density top band of 2*45*(am66), and (H and I) CsCl buoyant density bottom band of 2*45*(am66).

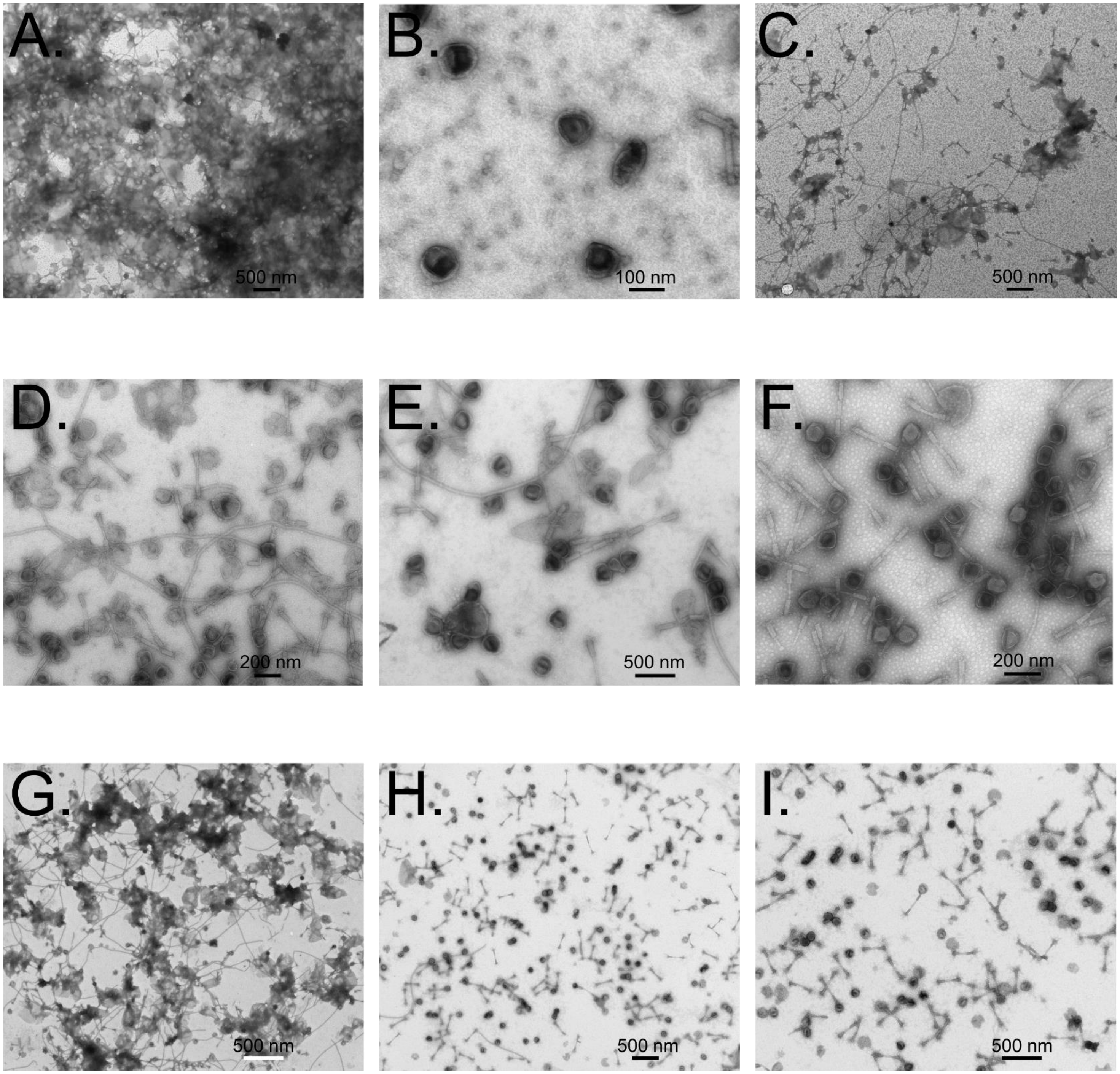

Figure S2. SDS-PAGE and DNA gels of purified *Salmonella* phage SPN3US protease mutants *245*(am59) and *245*(am66) grown under non-amber suppressing conditions. **(A)** Example CsCl step gradient banding profile of *245*(am59). **(B)** SDS-PAGE of the mutant *245*(am59) in comparison to the profile produced by the mutant purified after growth under amber suppressing conditions (am59-supF). Gel polyacrylamide concentration was 12 %. **(C)** Example CsCl buoyant density gradient banding profile of *245*(am59). **(D)** SDS-PAGE of buoyant density gradient bands from *245*(am59) and *245*(am66), compared to purified wild-type (WT) phage. Gel polyacrylamide concentration was 4-12 %. (**E and F**) Agarose gel electrophoresis of the samples of CsCl purified *245*(am59) and *245*(am66) from (B) and (D) versus WT phage and am107 (am107 forms DNA full heads by TEM, data not shown). Gels contained 1 % agarose in TAE buffer. Key: D, sample underwent differential concentration; DD, differentially concentrated sample underwent treatment with DTSSP; B, sample underwent CsCl buoyant density purification; BT, buoyant density gradient top band, BB, buoyant density gradient bottom band; M, Gene Ruler 1 kb DNA ladder (Thermo Scientific), 1 μg of total DNA was loaded per lane.

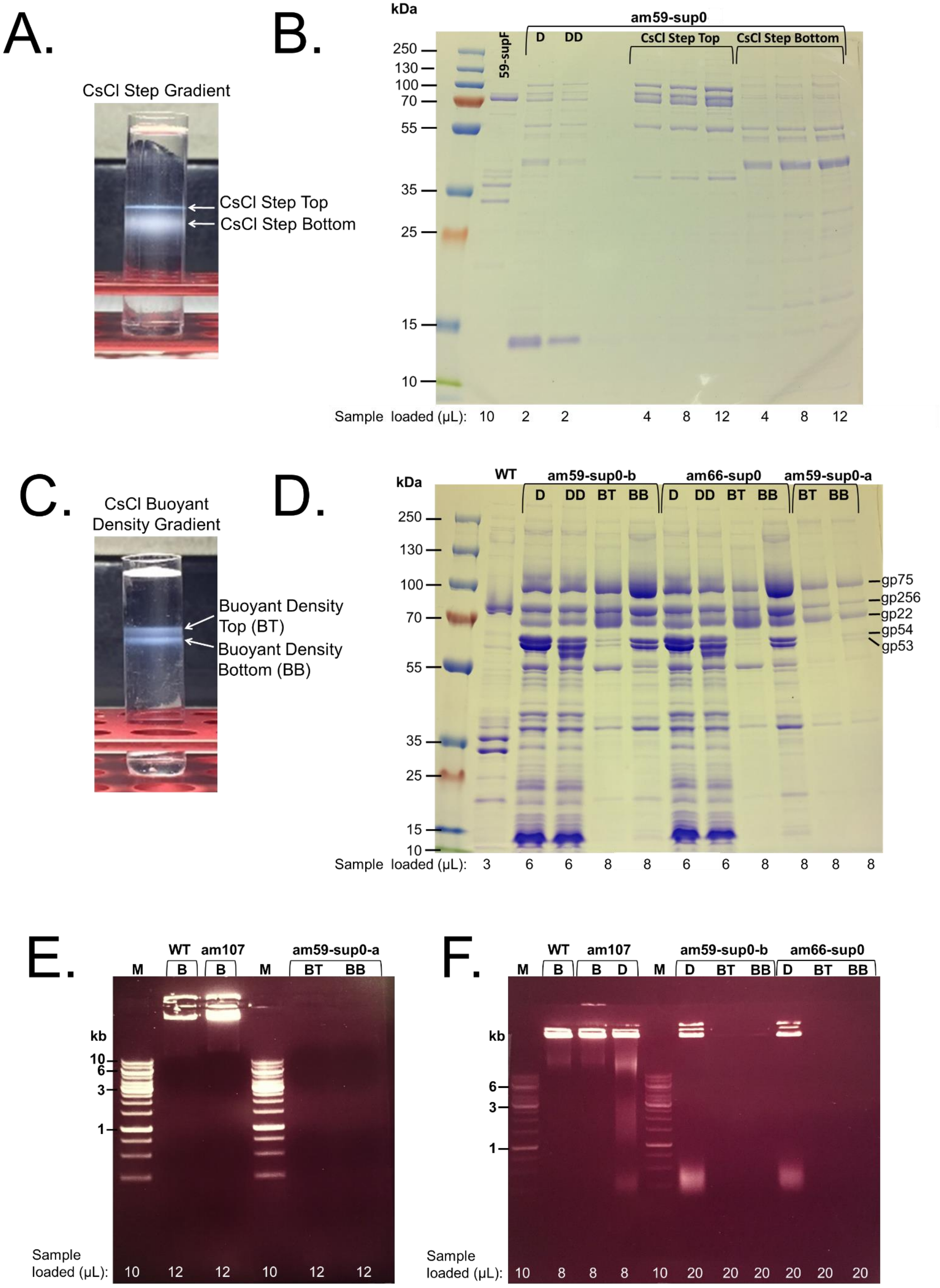
A.
CsCl Step Gradient
CsCl Step Top
CsCl Step Bottom
B.
kDa
250
130
100
70
55
35
25
15
10
59-supF
am59-sup0
D
DD
CsCl Step Top
CsCl Step Bottom
Sample loaded (μL): 10 2 2 4 8 12 4 8 12
C.
CsCl Buoyant Density Gradient
Buoyant Density Top (BT)
Buoyant Density Bottom (BB)
D.
kDa
250
130
100
70
55
35
25
15
10
WT
am59-sup0-b
am66-sup0
am59-sup0-a
D DD BT BB D DD BT BB BT BB
gp75
gp256
gp22
gp54
gp53
Sample loaded (μL): 3 6 6 8 8 6 6 8 8 8 8
E.
WT
am107
am59-sup0-a
M B B M BT BB
kb
10
6
3
1
Sample loaded (μL): 10 12 12 10 12 12
F.
WT
am107
am59-sup0-b
am66-sup0
M B B D M D BT BB D BT BB
kb
6
3
1
Sample loaded (μL): 10 8 8 8 10 20 20 20 20 20 20
